# Epilepsy characteristics and outcomes in patients with pleomorphic xanthoastrocytomas

**DOI:** 10.1007/s11060-026-05607-2

**Published:** 2026-06-19

**Authors:** Cody L. Nathan, Dina Ghandour, Elizabeth M. Cunningham, Rimas V. Lukas, Jared Sullivan, Sean Sachdev, Jessica W. Templer

**Affiliations:** 1 Epilepsy Division, Department of Neurology, Northwestern Memorial Hospital, 19th Floor, 259 E Erie St., Chicago, IL 60611 USA; 2 Oncology Division, Department of Neurology, Northwestern Memorial Hospital, Chicago, IL USA; 3 Lou and Jean Malnati Brain Tumor Institute at Northwestern Medicine, Chicago, IL USA; 4 Department of Radiation Oncology, Northwestern Memorial Hospital, Chicago, IL USA

**Keywords:** Pleomorphic xanthoastrocytoma, PXA, Tumor-related epilepsy, Electrocorticography, Circumscribed glioma

## Abstract

**Background:**

Pleomorphic xanthoastrocytomas (PXAs) are rare brain tumors frequently associated with tumor-related epilepsy. Few studies have analyzed epilepsy outcomes in relation to tumor characteristics, clinical features, or neurophysiologic findings in this patient population.

**Methods:**

This is a retrospective study of 25 patients diagnosed with a PXA at a single institution between 2004 and 2025. Clinical, pathologic, demographic, and treatment data were reviewed to identify factors associated with seizure outcomes after surgical resection.

**Results:**

Most patients (19/25, 76%) had tumor-related epilepsy with 13/19 (68.4%) presenting with seizure as the initial symptom. In retrospect, 6/19 (31.6%) patients had unrecognized focal seizures prior to the index presentation. After the first tumor resection prior to tumor recurrence, 15/19 (78.9%) patients were seizure free. At the most recent follow-up visit, 13/19 (68.4%) patients were seizure free. Seizure freedom at the most recent follow up was observed in 7/8 (87.5%) patients with ATRX loss compared with 1/5 (20%) without ATRX loss (*p* = 0.01). Of the patients with seizure recurrence, 5/6 (83.3%) had recurrence of tumor on MRI within 30 days of the breakthrough seizure (*p* = 0.003).

**Conclusions:**

Most patients with PXA-associated tumor-related epilepsy achieved seizure freedom after initial resection. Seizure recurrence was strongly associated with tumor recurrence at long-term follow up. ATRX loss was associated with seizure freedom at the most recent follow up, suggesting that tumor molecular features may help prognosticate epilepsy outcomes. Nearly one-third of patients had previously unrecognized focal seizures highlighting the importance of a detailed, anatomically guided seizure history.

## Key points

• Most PXA patients achieve seizure freedom after tumor resection.

• Seizure recurrence can strongly predict tumor recurrence.

• One-third of patients had unrecognized focal seizures before diagnosis of tumor.

## Introduction

Pleomorphic xanthoastrocytoma (PXA) is a rare, circumscribed central nervous system (CNS) tumor with moderately aggressive natural history (World Health Organization grade 2 or 3) [1, 2]. The mean age of diagnosis is in the late 20s but does occur across a wide age spectrum [1]. The 5-year overall survival is greater than 75%, and most cases that are WHO grade 2 can be treated with surgery alone [3]. Seizure is the most common presenting symptom, reported in 30–77% of cases at time of diagnosis [2, 4]. Other symptoms include elevated intracranial pressure or focal deficits dependent on tumor location [2]. The most common location for a PXA is the temporal lobe [2].

Data regarding seizure outcomes in the setting of PXA is limited. The largest and most recent study, by Zhou et al., reported that 17/27 (65%) of patients were seizure free after the initial tumor resection and before any tumor recurrence [4]. Patients who became seizure-free after initial resection had a younger age at seizure onset, smaller tumor diameter, and more likely to have BRAF-mutated tumors. This aligns with the growing understanding of the role of specific molecular characteristics in risk of tumor related epilepsy [5]. However, when accounting for tumor recurrence and the most recent tumor resection, these clinical features did not impact seizure freedom. Other studies report seizure freedom rates ranging from 63% to 90% [6, 7].

To our knowledge, few studies to date have assessed seizure outcomes in patients with PXAs who were evaluated by an epileptologist in addition to other members of their multidisciplinary care team (i.e. neuro-oncologist, neurosurgeon, radiation oncologist, etc.). Impact of electroencephalography (EEG) and/or intraoperative electrocorticography (ECoG) in prognostication of seizure outcomes is limited to case reports or series [6, 8, 9].

In this study, we aim to explore clinical, pathologic, and EEG features that may impact seizure outcomes in patients with PXAs and tumor-related epilepsy. We also aim to better understand the seizure semiologies (i.e., clinical signs and symptoms occurring during the seizure), EEG findings, ECoG findings, and seizure treatment patterns in this patient cohort.

## Methods

Patients with pathologically confirmed PXA were identified through the Northwestern University Nervous System Tumor Bank between 2004 and 2025, a period over which the WHO CNS Tumor Classification evolved. Of those patients with PXA, the chart was further reviewed for documentation of seizures at some point during their time course. A final cohort of patients with PXA and tumor-related epilepsy (TRE) was identified for further analysis. This study was approved by the institutional review board.

Demographic, clinical, pathologic, and treatment data in the electronic medical record were reviewed by an epileptologist. The index presentation was defined as the initial clinical presentation during which an abnormal lesion was identified on brain MRI. The extent of the surgical resection was categorized into gross total resection (100% of visible tumor removed), near-total resection (at least 90% of tumor removed but with some residual tissue), or subtotal resection (less than 90% of tumor removed) based on the operative note and corroborated by radiographic analysis.

Patients were defined as having TRE if the individual had a seizure at any timepoint during their disease course with evidence of seizure onset involving the region of the tumor (based on EEG and/or semiology) [10]. Seizure semiology was classified based on Luders et al. classification system [11]. Seizure frequency was quantified based on the American Academy of Neurology Seizure Frequency Process and Outcome Quality Measures [12]. Patients were determined to have suspected unrecognized seizures prior to the index presentation if the provider documented transient events that by history were consistent with seizures. History was also corroborated by reviewing routine EEGs, ambulatory EEGs, and/or continuous video EEG reports. Note, seizures occurring within 7 days of surgery were excluded to eliminate those patients with post-operative symptomatic seizures.

Seizure outcomes were divided into two categories: completely seizure free (Engel 1 A) or continued seizures [13]. The seizure outcomes were determined at two distinct timepoints. Timepoint 1 was defined as the latest time after the patient underwent initial tumor resection and prior to any known tumor recurrence on imaging. Timepoint 2 was defined as the most recent follow-up visit occurring after the patient’s most recent tumor resection and prior to any MRI scan noting interval change in tumor. Follow-up visits with neuro-oncology, oncology, epilepsy, neurosurgery, or radiation oncology were included in review. Seizure recurrence is defined as a documented seizure after having been seizure free for at least 30 days since surgery.

Data were stored in a password protected REDCap server. Descriptive statistics were reported using Stata [Version 19, StataCorp LLC, College Station, TX]. Categorical independent variables, such as genetic tumor markers and their impact on seizure outcome, were analyzed with a Fisher exact test. Independent variables with a continuous variable such as average patient’s age, tumor size, and impact on seizure outcome were analyzed using a two-tailed t-test in Stata.

## Results

A total of 25 patients were identified as having pathologically confirmed PXA. Of these 25 patients, 19 (76%) had tumor-related epilepsy based on documentation in the electronic medical record. Data analysis focused primarily on those with TRE; patients with PXA but without TRE were excluded. The majority of patients with TRE were female (12/19, 63.2%), and the mean age at diagnosis was 32 years (range 10–71 years). The mean duration of follow up was 7.25 years (range 0.68–23.6). The remainder of patient demographic information is listed in Tables 1 and 2 summarized individualized patient data.

**Table 1:** Demographics, patient characteristics, seizure outcomes

Variable	Total	Before Tumor Recurrence (Timepoint 1)		P-value	After last resection		P-value
					(Timepoint 2)		
		Seizure free	Not seizure free		Seizure free	Not seizure free	
**Total**		15	4		13	6	
**Gender**							
Male	7/19 (36.8%)	5	2	0.60^a^	6	1	0.33^a^
Female	12/19 (63.2%)	10	2		7	5	
**Race**							
White	13/19 (68.4%)	9	4	0.51^a^	8	5	0.68^a^
Asian	2/19 (10.5%)	2	0		1	1	
Black	0 (0%)	0	0		0	0	
America Indian	0 (0%)	0	0		0	0	
Unknown	2/19 (10.5%)	3	0		2	0	
Declined	2/19 (10.5%)	2	0		2	0	
**Ethnicity**							
Hispanic/Latino	2/19 (10.5%)	1	1	0.65^a^	2	0	0.36^a^
Non-Hispanic	17/19 (79.0%)	12	3		9	6	
Declined	2/19 (10.5%)	2	0		2	0	
**Mean age at diagnosis** (range)	32 years (SD = 15.0) (Range 10-71)						
		34	26	0.81^b^	32	31	0.55^b^
		(SD = 16)	(SD = 9)		(SD= 16)	(SD= 14)	
**Tumor laterality**							
Right	7/19 (36.8%)	6	1	0.58^a^	6	1	0.33^a^
Left	12/19 (63.2%)	9	3		7	5	
**Tumor Location**							
Frontal	5/19 (26.3%)	4	1	0.99^a^	5	0	0.09^a^
Temporal	9/19 (47.4%)	7	2		4	5	
Parietal	5/19 (26.3%)	4	1		4	1	
Occipital	0 (0%)	0	0		0	0	
Other/brainstem	0 (0%)	0	0		0	0	
**Metastasis **							
Yes	4/19 (21.0%)	2	2	0.18^a^	3	1	0.75^a^
No	15/19 (79.0%)	13	2		10	5	
**Tumor Grade**							
WHO 1	0 (0%)	0	0	0.53^a^	0	0	0.71^a^
WHO 2	3/19 (15.8%)	2	1		2	1	
WHO 3	16/19 (84.2%)	13	3		11	5	
**Initial Resection Type**							
Gross Total							
Near Gross Total or Subtotal	11/19 (57.9%)	7	4	0.10^a^	7	4	0.49^a^
	8/19 (42.1%)	8	0		6	2	
**BRAF V600E mutation**							
Yes							
No	14/19 (73.7%)	10	4	0.62^a^	8	6	0.33^a^
Unknown	1/19 (5.2%)	1	0		1	0	
	4/19 (21.0%)	4	0		4	0	
**CDKN2A or CDKN2B deletion **							
Yes							
No							
Unknown	13/19 (68.4%)	9	4	0.43^a^	7	6	0.16^a^
	1/19 (5.3%)	1	0		1	0	
	5/19 (26.3%)	5	0		5	0	
**TERT promoter mutation**							
Yes							
No	5/19 (26.3%)	3	2	0.25^a^	2	3	0.39^a^
Unknown	7/19 (36.8%)	5	2		5	2	
	7/19 (36.8%)	7	0		6	1	
**ATRX loss**							
Yes	5/19 (26.3%)	3	2	0.63^a^	1	4	0.01^a*^
No	8/19 (42.1%)6/19 (31.6%)	7	1		8	0	
Unknown		5	1		4	2	
**IDH1 or IDH2 mutation**							
Yes							
No	1/19(5.2%)	0	1	0.30^a^	0	1	0.18^a^
Unknown	15/19 (79.0%)	12	3		10	5	
	3/19 (15.8%)	3	0		3	0	
**GFAP mutation**							
Yes	18/19 (94.8%)	14	4	0.79^a^	12	6	0.68^a^
No	0/19 (0%)	0	0		0	0	
Unknown	1/19 (5.2%)	1	0		1	0	
**Olig2 mutation**							
Yes	7/19 (36.8%)	5	2	0.76^a^	0	1	0.53^a^
No	1/19 (5.2%)	1	0		5	2	
Unknown	11/19 (58.0%)	9	2		8	3	
**MGMT promoter methylated**							
Yes							
No							
Unknown	6/19 (31.6%)	4	2	0.48^a^	4	3	0.21^a^
	7/19 (36.8%)	5	2		3	3	
	6/19 (31.6%)	6	0		6	0	
**Radiation post surgery**							
Yes							
No	15/19 (79.0%)	12	3	0.65^a^	10	5	0.63^a^
	4/19 (21.0%)	3	1		3	1	
**Seen by epileptologist**							
Yes							
No	8/19 (42.0%)	5	3	0.26^a^	6	2	0.49^a^
	11/19 (58.0%)	10	1		7	4	
**Presenting Seizure Type**							
Focal seizure							
Bilateral tonic-clonic seizure	6/13 (46.2%)	5	1	0.46	3	3	0.27^a^
	7/13 (53.8%)	7	0		6	1	
**ECoG Guided Resection**							
Yes							
No	1/19 (5.2%)	1	0	0.43^a^	1	0	0.51^a^
	18/19 (94.8%)	11	7		11	7	
**Status epilepticus**							
Yes							
No	1/19 (5.3%)	1	0	0.80^a^	12	6	0.68^a^
	18/19 (94.7%)	14	4		1	0	
**Greatest tumor volume** (cm^3^) at initial imaging, mean (SD)	37.12						
	(SD 37.51) (Range 1.58-138.30)	30.1	56.45	0.24^b^	36.7	38.3	0.95^b^
		(SD=39.44)	(SD=26.45)		(SD=140.74)	(SD=32.1)	
**Greatest FLAIR volume **(cm^3^) at initial imaging, mean (SD)	29.11						
	(SD 31.88)	21.18	50.93	0.11^b^	29.85	27.08 (SD= 33.09)	0.88^b^
	(Range 0.94-104.49)	(SD = 30.34)	(SD = 28.46)		(SD = 33.05)		
**Greatest enhancing tumor volume** (cm^3^) at initial imaging, mean (SD)	8		5.52 (SD =6.27)				
	(SD 9.06)	8.92		0.54^b^	6.84 (SD = 10.44)	11.22 (SD = 1.17)	0.42^b^
	(Range 0-33.8)	(SD=					
		9.99)					

^a^ Fisher exact test

^b^ Two-tailed t-test

^*^Statistically significant (p<0.05)

**Table 2:** Individualized patient information

Patient	Gender	Race	Ethnicity	Age at diagnosis	Tumor laterality	Tumor location	Metastasis	Tumor Grade	Initial resection type	BRAF V600E mutation	ATRX loss	CDKN2A or CDKN2B deletion	IDH1 or IDH2 mutation	GFAP mutation	Olig2 mutation	MGMT promotor methylates	Presenting Symptom	ECoG Guigui resection	Status epilepticus	Seen by epileptologist	Seizure free Timepoint 1	Seizure free Timepoint 2
1	F	Asian	Non-Hispanic	32	Left	Temporal	No	WHO III	STR	Yes	Unknown	Yes	No	Yes	No	Yes	FAS	No	No	No	No	No
2	F	White	Non-Hispanic	23	Left	Frontal	No	WHO II	GTR	Yes	No	Yes	No	Yes	Yes	Yes	FTBCS	Yes	No	Yes	Yes	Yes
3	F	White	Non-Hispanic	30	Left	Parietal	No	WHO II	GTR	Yes	No	Yes	No	Yes	Yes	No	Headache	No	No	Yes	Yes	Yes
4	F	White	Non-Hispanic	19	Right	Temporal	No	WHO III	STR	Yes	Yes	Yes	No	Yes	Unknown	No	FIAS	No	No	No	No	No
5	F	Asian	Non-Hispanic	25	Right	Temporal	Yes (leptomeninges)	WHO III	NGTR	Unknown	Unknown	Unknown	Unknown	Yes	Unknown	Unknown	FTBTCS	No	No	Yes	Yes	Yes
6	F	White	Non-Hispanic	21	Left	Temporal	Yes (pulmonary)	WHO III	GTR	Yes	Yes	Yes	No	Yes	Yes	No	FIAS	No	No	Yes	No	Yes
7	F	White	Non-Hispanic	20	Left	Parietal	No	WHO III	NGTR	Unknown	Unknown	Unknown	No	Yes	Unknown	Unknown	FTBTCS	No	No	No	Yes	Yes
8	F	White	Non-Hispanic	21	Right	Parietal	Yes (dura)	WHO III	GTR	Yes	No	Yes	No	Yes	Yes	No	Headache	No	No	No	Yes	Yes
9	F	White	Non-Hispanic	43	Left	Temporal	No	WHO III	GTR	Yes	Yes	Yes	No	Yes	Yes	Yes	FTBTCS	No	No	No	No	No
10	M	White	Non-Hispanic	21	Left	Temporal	No	WHO II	GTR	Yes	Unknown	Yes	Yes	Yes	Unknown	Yes	Asymptomatic	No	No	Yes	No	No
11	F	White	Non-Hispanic	52	Left	Parietal	No	WHO III	GTR	Yes	Yes	Yes	No	Yes	Unknown	No	Dizziness	No	No	No	Yes	No
12	M	Unknown	Non-Hispanic	24	Left	Temporal	No	WHO III	GTR	Yes	No	Yes	No	Yes	Yes	Yes	FTBTCS	No	No	Yes	Yes	No
13	M	White	Non-Hispanic	52	Right	Frontal	No	WHO III	NGTR	Unknown	Unknown	Unknown	Unknown	Yes	Unknown	Unknown	FAS	No	No	No	Yes	Yes
14	M	White	Hispanic or Latino	41	Left	Frontal	No	WHO III	GTR	Yes	Yes	Yes	No	Yes	Unknown	Yes	Dizziness	No	No	Yes	Yes	Yes
15	M	Declined	Declined	23	Right	Parietal	No	WHO III	STR	No	No	Unknown	No	Yes	Unknown	Unknown	FTBTCS	No	No	No	Yes	Yes
16	F	Unknown	Hispanic or Latino	44	Right	Temporal	No	WHO III	GTR	Yes	No	Yes	No	Yes	Unknown	No	FTBTCS	No	No	No	Yes	Yes
17	F	White	Non-Hispanic	35	Right	Frontal	No	WHO III	GTR	Yes	No	No	No	Yes	Yes	No	FAS	No	No	Yes	Yes	Yes
18	M	Declined	Declined	10	Left	Temporal	Yes (spine)	WHO III	STR	Yes	No	Yes	No	Yes	Unknown	Unknown	Blurry vision	No	Yes	No	Yes	Yes
19	M	White	Non-Hispanic	71	Left	Frontal	No	WHO III	NGTR	Unknown	Unknown	Unknown	Unknown	Unknown	Unknown	Unknown	FIAS	No	No	No	Yes	Yes

GTR: Gross total resection; NGTR: Near gross total resection; STR: Subtotal resection; FAS: Focal aware seizure; FIAS: Focal impaired aware seizure; FTBCS: Focal to bilateral tonic-clonic seizure; BTCS: Bilateral toinc-clonic seizure

The most frequent tumor location for patients with TRE was the temporal lobe (9/19, 47.4%) followed by frontal (5/19, 26.3%), and parietal (5/19, 26.3%). The location of the tumor had no impact on seizure outcome at either timepoint (*p* = 0.99 and 0.09, as listed in Table 1). The mean initial tumor volume was 37.4 cm^3^ (range 1.6-138.3 cm^3^), FLAIR tumor volume was 27.9 cm^3^ (range 0.9-104.5 cm^3^), and enhancing volume 9.6 cm^3^ (range 0-42.5 cm^3^). Higher mean initial total volume and FLAIR volume trended towards being associated with worse seizure outcomes though this was not statistically significant (Table 1). For the initial surgery, gross total resection was performed on 11/19 (57.8%) of patients, near gross total on 4/19 (21.1%) patients, and subtotal resection on 4/19 (21.1%) of patients. Patients who did not undergo a gross total resection had tumor near eloquent cortex or tumor was near an area of at-risk vasculature. The initial resection type did not significantly impact seizure outcome at either timepoint. There were 14/19 (73.7%) patients who had tumor recurrence during their clinical course, though initial resection type (gross total resection compared to near gross total or subtotal resection) did not significantly impact tumor recurrence rates (*p* = 1.0). The remainder of tumor characteristics, including genetic markers, are outlined in Table 1.

The median time from initial imaging finding to first surgery was 9 days (range 0–5,475 days). The patient who did not go for surgery for 5,475 days was initially diagnosed with a lesion in 2006 but was then lost to follow up until 2019 at which time care was re-established. A total of 13/19 (68.4%) patients required a second surgery due to tumor recurrence. The median time from the first surgery to second surgery was 1.93 years (range 0.06-11.91). A third surgery was required for 4/19 (21.1%) patients due to tumor recurrence. The median time from the second surgery to a third surgery was 4.26 years (range 2.29–15.11 years). There was only 1/19 (5%) patient who underwent chemotherapy prior to the initial resection, whereas 15/19 (78.9%) underwent chemotherapy post-initial resection. A total of 15/19 patients (78.9%) underwent radiation treatment post-resection. Chemotherapy and radiation had no impact on seizure outcomes at either timepoint (Table 1).

**Fig. 1:**
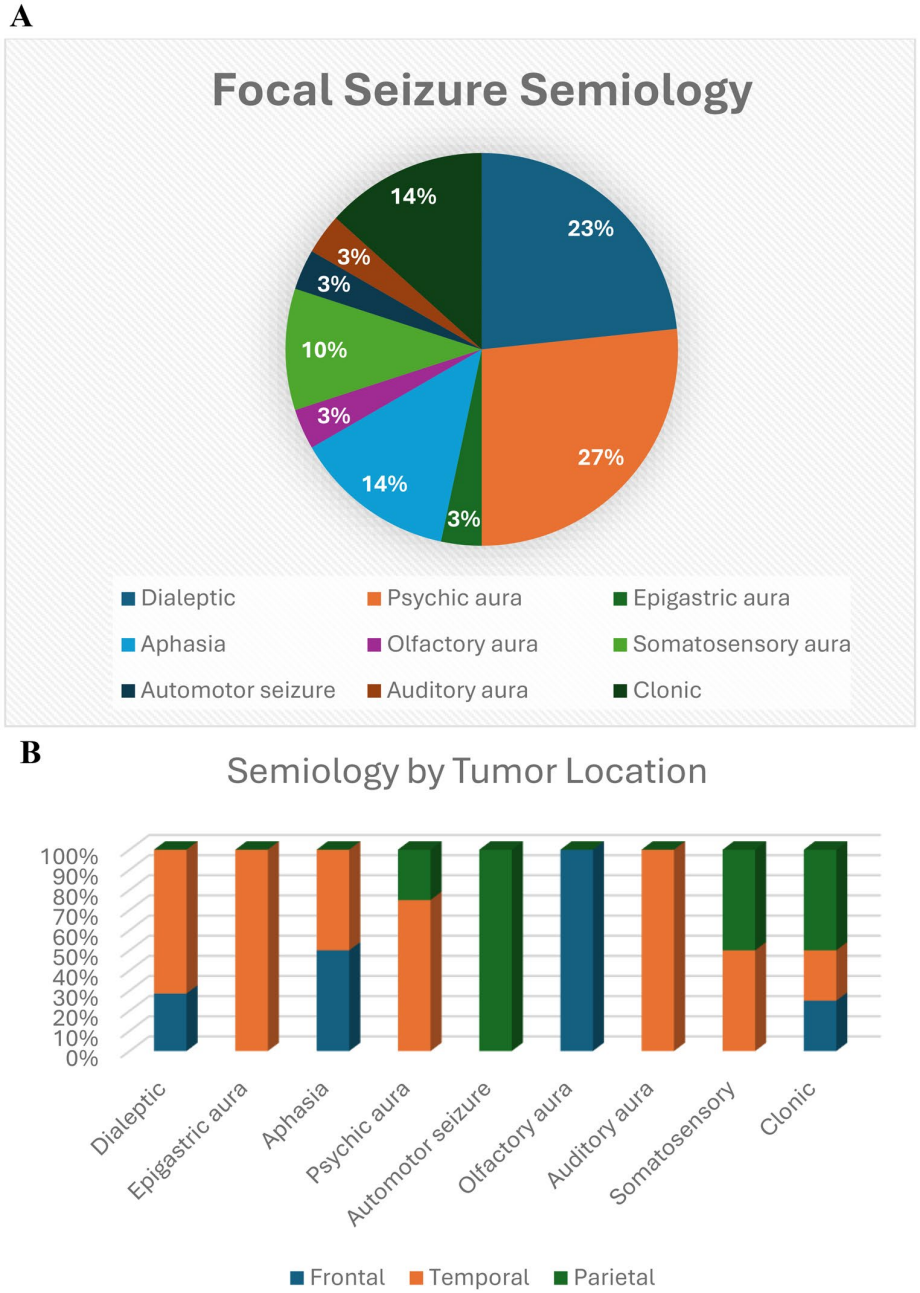
**A** Distribution of focal seizure semiology amongst patients. **B** Seizure semiology based on tumor location. Definitions of the above-mentioned terminology regarding seizure/aura types [11]. Semiology: Observable clinical signs and symptoms of the seizure. Dialeptic: Seizure with alteration of consciousness. Aphasic: Sudden language impairment. Automotor: Complex motor movements that can resemble natural movements but occur in an inappropriate setting. Psychic aura: Complex hallucinations or illusions that affect different senses. Auditory aura: Illusions/hallucinations of sounds. Epigastric aura: Abdominal sensations. Somatosensory aura: Abnormal paresthesia limited to a defined region of the body. Clonic: Muscle contractions at a regular rate of 0.2-5 movements per second

**Fig. 2:**
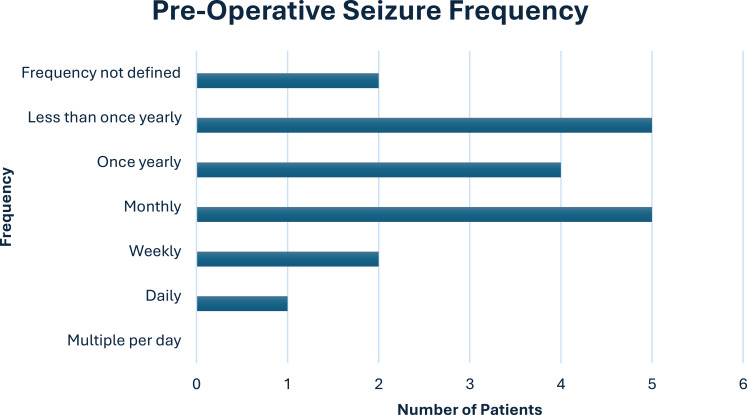
Pre-operative seizure frequency

More than two-thirds of patients with TRE (13/19, 68.4%) presented with a seizure as the initial symptom and the others presented with symptoms including headache, blurred vision, and dizziness. At initial presentation, the distribution of seizure type was comparable between focal seizures (6/13, 46.2%) and focal to bilateral tonic-clonic seizures (7/13, 53.8%). The most common ongoing focal seizure semiologies were psychic auras (focal aware seizures) and dialeptic seizures (focal impaired aware seizures) as seen in Fig. 1A. Seizure semiology based on tumor location is depicted in Fig. 1B. Presenting seizure type did not impact seizure freedom at either timepoint. There were two patients who did not have any seizures until after their first surgery. Preoperative seizure frequency varied (Fig. 2), and there was no statistically significant difference in seizure outcomes at either timepoint based on seizure frequency.

According to detailed histories, 6/19 (31.6%) patients with TRE had suspected unrecognized focal seizures prior to their initial imaging finding and diagnosis of a PXA. It was only during their history taking by an epileptologist (4/6, 66.6%) or a neuro-oncologist (2/6, 33.3%) that episodes occurring prior to their diagnosis were identified as unrecognized seizures. Most patients (5/6, 83.3%) with previously unrecognized seizures were experiencing psychic auras characterized by symptoms including anxiety, depersonalization, or déjà vu. One patient also had an associated olfactory aura (sudden perception of smell that is not present in the environment). For all six patients, the patients initially had retained awareness but then progressed to loss of awareness, though only 2/6 patients (33.3%) ever went on to have a bilateral tonic-clonic seizure. Half of the patients with unrecognized seizures (3/6, 50%) had tumors in the temporal lobe; 2/6 (33.3%) had frontal lobe tumors, and 1/6 (16.7%) had a parietal lobe tumor. Among the 13/19 patients who presented with a seizure as the initial symptom, five had suspected unrecognized seizures even prior to that initial presentation.

The most commonly prescribed anti-seizure medication (ASM) before the initial resection and perioperatively was levetiracetam (12/19, 63.2%, preoperatively, 14/19, 73.7%, perioperatively). Other less commonly prescribed ASMs used during the disease course included oxcarbazepine, phenytoin, lacosamide, clobazam, lamotrigine, and perampanel.

At timepoint 1 (i.e., after initial resection), 15/19 (78.9%) of patients were seizure free (Engel 1 A). There was no statistically significant difference in outcome based on WHO grade. A total of 11/15 (73.3%) patients who were seizure free at timepoint 1 remained seizure free at the most recent follow up visit (Table 1). Of the eleven patients who remained seizure free after the initial resection, eight continued the same ASM regimen after surgery whereas three patients had ASMs discontinued. All patients with ASMs discontinued had no evidence of tumor recurrence at time of wean. Anti-seizure medication was weaned between 3 and 12 months after initial surgery.

Four of fifteen (26.7%) patients who were seizure free after their initial resection later experienced seizure recurrence. Two out of these four (50%) patients were found to have tumor recurrence on MRI obtained within 30 days of seizure recurrence. The two patients with seizure recurrence in the context of tumor recurrence initially had a gross total resection whereas the two with seizure recurrence in the absence of imaging changes had a subtotal resection.

At the most recent follow up following the latest tumor resection (timepoint 2), 13/19 (68.4%) patients remained seizure free. There was no statistically significant difference in seizure outcome and WHO grade (Table 1). Of the six patients with seizure recurrence since their most recent resection, 5/6 (83.3%) had recurrence of tumor on MRI within 30 days of the reported breakthrough seizure which is significant compared to those patients who were seizure free (*p* = 0.003). Of these same patients with seizure recurrence, anti-seizure medications had been weaned in 1/6 (16.7%) of patients, while 5/6 (83.3%) of patients were either on their same dose of antiseizure medications, a higher dose, or on additional antiseizure medications. There was no statistically significant difference between seizure recurrence if anti-seizure medications were weaned.

All patients with ATRX loss were seizure free at the last follow-up visit. However, if ATRX expression was maintained, patients were more likely to have continued seizures (4/5, 80%). ATRX loss was significantly associated with seizure freedom (*p* = 0.01). Of note, the status of ATRX expression was unknown in six patients. The remainder of tumor genetic markers (outlined in Table 1) had no statistically significant impact on seizure freedom.

A small proportion of all patients underwent an EEG preoperatively (6/19, 31.6%) with 3/6 (50%) of these EEGs showing epileptiform activity. All 3 of the patients with pre-operative EEG epileptiform activity were seizure free after their initial resection (i.e., timepoint 1). Preoperative EEG epileptiform activity did not have a significant impact on seizure outcome. Postoperatively, 11/19 (57.9%) patients underwent an EEG. The 6/11 (54.5%) EEGs without any epileptiform activity all went on to be seizure free throughout the remainder of their disease course. Of the five patients with epileptiform activity, 3/5 (60%) continued having seizures at both timepoint 1 and 2.

**Fig. 3:**
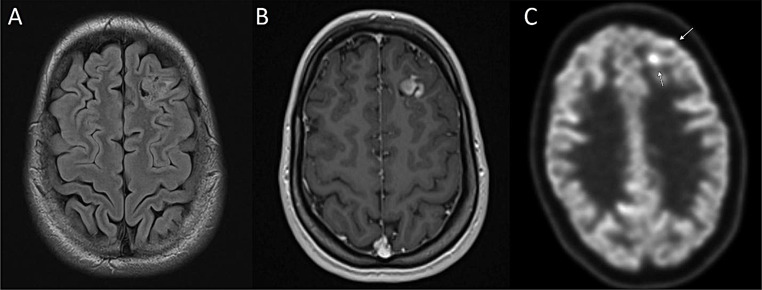
Patient imaging. A 24-year-old woman who initially presented with a bilateral tonic-clonic seizure and found to have a left frontal enhancing lesion. Panel **A** shows an axial T2 FLAIR image showing a 1.8 x 1.5 x 2.3 cm lesion within the cortex bridging the left superior frontal sulcus. Panel **B** showed axial T1 post-contrast imaging showing a 2.3 cm heterogeneously enhancing lesion centered along the cortex bridging the midportion of the left superior frontal sulcus. Panel **C** shows PET CT scan with focal hypermetabolic activity in the left anterior frontal lobe corresponds to the enhancing components of the 2.3 cm lesion in this location on prior brain MRI (arrow). Focal decreased activity just superior to the above-described region in the left frontal lobe cortex (arrow)

**Fig. 4:**
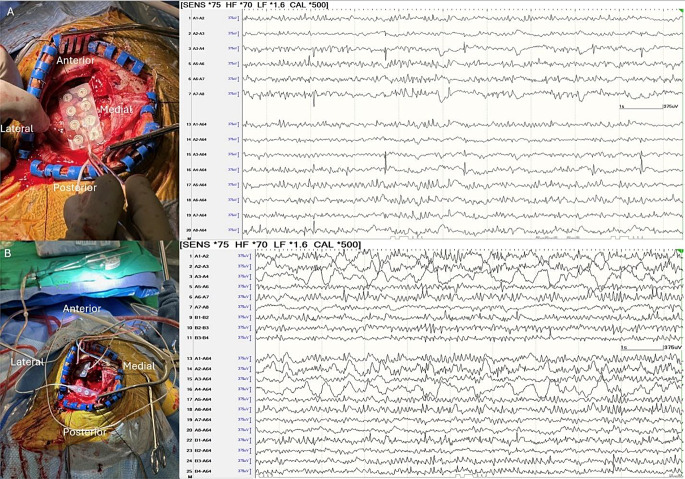
Patient intraoperative ECoG. Patient underwent a craniotomy with ECoG guided resection. Panel **A** shows pre-resection ECoG with one 2x4 grid over position 1 showed spikes over the posterior contact A4 and A6. Panel **B** shows ECoG after gross total resection with one 2x4 grid and one 1x4 strip over the resection cavity ECoG with no appreciated epileptiform activity

Only 1/19 (5.3%) patient underwent intraoperative ECoG guided resection for a left frontal PXA (Fig. 3). Epileptiform discharges were recorded with pre-resection ECoG and resolved after gross total resection (Fig. 4). The patient remained seizure free after resection.

## Discussion

The majority of patients in our cohort had tumor-related epilepsy and seizure as the initial reported symptom, similar to previous literature regarding this topic [4]. Most patients were seizure free after their initial resection and remained seizure free in the absence of tumor recurrence. However, 21.1% of patients continued having seizures after initial resection. Of the remaining patients who were initially seizure free after resection, 26.7% (4/15) later had seizure recurrence. Imaging obtained in response to these seizures revealed tumor recurrence in half of these patients while the other half of patients had no tumor changes identified on imaging. Interestingly, both the patients with seizures recurring in the context of stable MRIs had previously undergone a subtotal resection. Perhaps an incomplete removal of the epileptogenic region with the initial surgery led to seizure recurrence.

Over two-thirds of the patients at the most recent clinic visit were seizure free since their most recent resection. Of the patients who were not seizure free, all but one was found to have recurrence of tumor on MRI within 30 days of the breakthrough seizure. In general, tumor recurrence was common in our cohort, with over half requiring a second surgery and nearly a quarter of the patients requiring a third surgery. Seizure recurrence significantly correlated with tumor recurrence over the entire disease course. Thus, suspicion for tumor recurrence should be considered for patients who were once seizure free but have a relapse in seizures.

Another key feature in this study is that nearly one-third of patients in this cohort had suspected seizures prior to their index presentation. This raises the possibility that additional patients with PXA may have undiagnosed tumor-related epilepsy due to events unrecognized as seizures. Identification of transient events as seizures can vary depending on the clinical features of the seizure (i.e. seizure semiology), patient reporting of information, and the clinician’s expertise [14]. The rates of misdiagnosis of epilepsy range from ~ 4–30% depending on the clinical setting. Misdiagnosis can happen in non-epilepsy specific clinics but also epilepsy clinics [14]. Bilateral tonic-clonic seizures and focal motor seizures are more easily recognized compared to nonmotor focal seizures [15]. In a retrospective study of 447 patients with epilepsy, the diagnosis of epilepsy in patients with nonmotor seizures compared to motor seizures was 10 times longer [15]. Of the patients in our cohort with unrecognized seizures, one-third had only focal seizures without progression to bilateral tonic-clonic seizures.

In our cohort, unrecognized seizures preceding the diagnosis of PXA were identified by an epileptologist in two-thirds of patients and by non-epilepsy physicians in the remaining one-third. Referral to an epileptologist can vary depending on referring provider comfort with managing epilepsy, complexity of epilepsy, and patient preference, among other potential reasons.

Due to the relative rarity of PXAs and thus small sample size, the study was underpowered to detect statistically significant associations between some assessed variables (i.e. BRAF mutations) and seizure freedom. Interestingly, seizure freedom at the last clinic visit was associated with ATRX loss. ATRX is a tumor suppressor gene involved in chromatin remodeling and the SWI/SNF complex. Data in terms of outcomes in patients with ATRX mutations and PXA tumors is limited to case reports as this is a relatively rare mutation. The limited cases of ATRX loss of expression mutation with PXA do not report on seizure incidence [16, 17].

Less than one-third of patients underwent a preoperative EEG, two of which were normal despite patients having a confirmed diagnosis of epilepsy. The finding highlights that a normal EEG does not rule out the diagnosis of epilepsy. Preoperative interictal epileptiform abnormalities did not impact the seizure outcome at either timepoint. Interestingly, all the patients with preoperative EEGs with epileptiform abnormalities became seizure free after the initial resection. This corroborates findings published in two case reports where both patients had epileptiform discharges on EEG preoperatively and ultimately were seizure free at 6 months and 22 months respectively [7, 8]. Postoperatively in our patient cohort, patients who had an EEG and were found to have ongoing epileptiform abnormalities tended not to be seizure free though this was not statistically significant. Larger retrospective studies looking at low-grade epilepsy-associated brain tumors found that epileptiform discharges on intraoperative ECoG and scalp EEG post-resection was a negative factor on postoperative seizure freedom at 1, 2, and 5 year follow up [18]. The study included 111 patients, 3 of which had a PXA, though specific outcomes of the patients with PXAs were not reported.

Only one patient in our cohort had intraoperative ECoG which showed peri-lesional epileptiform discharges pre-resection which were no longer seen after resection. This patient remains seizure free. A meta-analysis of 83 studies assessing seizure outcome after intraoperative ECoG-tailored epilepsy surgery noted favorable seizure outcomes particularly in patients with tumor-related epilepsy [19]. Patients with tumor-related epilepsy who underwent ECoG guided resection had a seizure freedom rate of 86% [19].

The major limitation of the study is the small sample size and single center retrospective design, which likely contributed to the lack of statistically significant findings. Only three patients successfully weaned off ASMs after surgery and remained seizure free at their most recent follow-up. The timing of when ASMs were weaned was highly variable, ranging from 3 months to 6 years after the initial surgery. A larger cohort of patients with PXA tumors would be necessary to discern which factors contribute to patients successfully weaning off ASMs and timing of weaning. An additional limitation is that a minority of the patients underwent a preoperative EEG. We can extrapolate from the 2025 clinical practice guidelines for the diagnosis and treatment of diffuse glioma-related epilepsy that an EEG is advised to confirm that seizures are concordant with a patient’s lesion [20]. While PXAs are circumscribed gliomas and not diffuse gliomas, EEG can aid in confirming the seizure onset zone for patients.

In summary, most patients with PXA-associated tumor-related epilepsy in our single center cohort achieved seizure freedom after initial resection. However, seizure recurrence was strongly associated with tumor recurrence at long-term follow up. ATRX loss was significantly associated with seizure freedom at the most recent follow up, suggesting that tumor molecular features may help prognostic epilepsy outcomes. Nearly one-third of patients had previously unrecognized focal seizures, most commonly psychic auras including déjà vu and fear, highlighting the importance of taking a detailed, anatomically guided history. 

## Data Availability

The datasets generated during and/or analyzed during the current study are available from the corresponding author on reasonable request.
